# Tofacitinib combined with local low-dose ixekizumab injection benefits those with peripheral psoriatic arthritis

**DOI:** 10.7555/JBR.37.20220253

**Published:** 2023-10-28

**Authors:** Ruiyuan Xia, Weixin Zhang, Jing Hang, Zhiqiang Yin

**Affiliations:** 1 Department of Dermatologythe First Affiliated Hospital of Nanjing Medical UniversityNanjing, Jiangsu 210029China; 2 Department of Ultrasoundthe First Affiliated Hospital of Nanjing Medical UniversityNanjing, Jiangsu 210029China

Dear Editor,

Treating psoriatic arthritis (PsA) is always difficult. Systemic treatments can be administered either orally or through intramuscular and intra-articular injection, including conventional synthetics, biologics and targeted synthetic disease-modifying antirheumatic drugs^[1]^. The alternatives, topical external therapies, are not effective on joint lesions due to drug permeability issues. Drugs injected into the articular cavity are also unsuitable for small peripheral joint lesions, the most common manifestations of PsA. The limited treatment options for PsA present a challenge.

This is a case of a 26-year-old woman with a six-year history of psoriasis vulgaris, who had been suffering from co-morbid PsA for four years. The case presented at our clinic six months ago with PsA in multiple interphalangeal joints. The skin lesions were distributed on her hands, elbows and knees, which were light red, raised from the surface and covered with fine scales, accounting for about 25% of the upper limbs and 8% of the lower limbs. The Psoriasis Area and Severity Index (PASI) was 2.4. On her nails, the main manifestations were pitting, onycholysis and splinter hemorrhages. The baseline Nail Psoriasis Severity Index (NAPSI) was 32. The scalp appeared unaffected. Laboratory investigations revealed negative results for rheumatoid factor and human leucocyte antigen B27.

Previous topical steroid treatments combined with nonsteroidal anti-inflammatory drugs and methotrexate had all failed to improve symptoms. The therapy with etanercept improved skin and joint lesions initially, but was not tolerated thereafter due to leukocytopenia. The case was administered tofacitinib, an oral Janus kinase inhibitor, at 5 mg, twice daily two years ago. This therapy improved skin symptoms and joint pain, but the joint redness and swelling lasted. The dose was reduced to 5 mg once daily a year ago due to concerns about potential infections. Consequent tofacitinib treatments stabilized joint symptoms with mild pain, but visible redness, swelling and malformation continued.

The patient was very concerned about the side effects of systemic medication, and her arthritis was mostly confined to a few small joints. The locally injected antibody may be more effective to the target site through direct diffusion or interstitial fluid movement, thus reducing costs and systemic side effects^[2]^. After full communication with the patient, we finally decided to combine local injections in the treatment.

**Figure 1:**
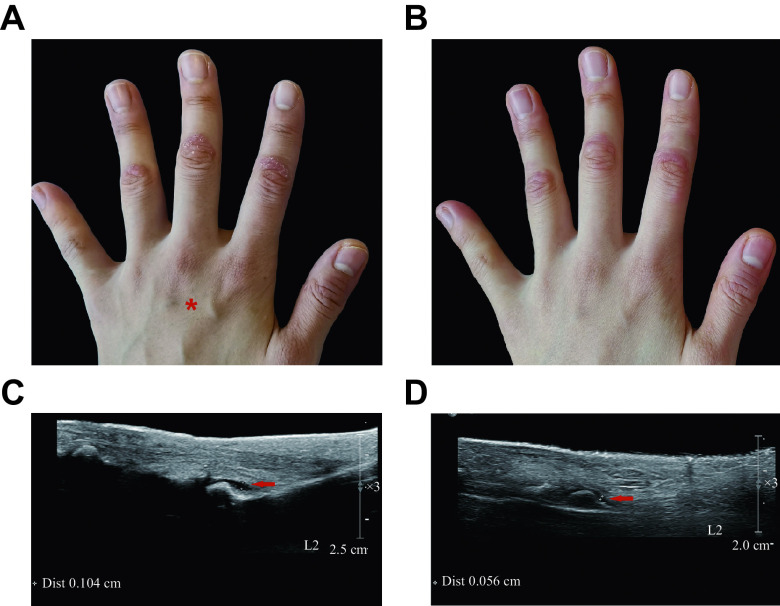
Before and after the combination therapy

After receiving the written informed consent from the patient, we began the treatment with low-dose ixekizumab (an IL-17 blockade) administered subcutaneously near the joint lesions to supplement the existing treatment, *i.e.*, daily tofacitinib at a dose of 5 mg. The back of her left hand (***Fig. 1A***) received a subcutaneous injection of 0.1 mL diluted ixekizumab (8 mg/mL) every two weeks. Ixekizumab (80 mg/mL) was diluted with sterile water to 8 mg/mL in the pharmacy intravenous admixture service, and stored at 4 ℃.

Two weeks after the treatment, both joint symptoms and skin lesions on the fingers of her left hand had improved (***Fig. 1B***). By week eight, the redness and swelling on proximal interphalangeal joints of her forefinger, middle finger and ring finger had substantially improved. The hydrops articuli of proximal interphalangeal joints on her left hand had markedly reduced after the eight-week treatment (***Fig. 1C*** and ***1D***). However, the hydrops articuli on her right fingers had not ameliorated. The only adverse event was transient but bearable pain, while injecting interventions. The patient reported a continued improvement in joint symptoms in her left hand at week 12.

The score of the visual analogue scale for the third and fourth left fingers decreased from three and two, respectively, to one and zero over the observation period. However, joint pain in her right hand was not ameliorated. The skin lesions of her left hand had improved; however, the lesions of other parts of the body remained the same and an overall PASI score stayed at 2.4. Her onycholysis and splinter hemorrhages had significantly improved, while nail pitting had not. The NAPSI score was 19 at week 12. The patient remains under a long-term monitoring.

Previously, we successfully used intramatrical injections with low-doses of secukinumab to treat nail psoriasis^[3–4]^ and local low-dose ixekizumab to treat palmoplantar pustular psoriasis^[5]^, which suggested the feasibility and efficacy of local low-dose biologics injection. The current case study also suggested that combining juxtra-articular low-dose ixekizumab injection with oral tofacitinib could benefit those with peripheral psoriatic arthritis. The subcutaneous injections administered near joints could well be absorbed by peripheral joint lesions, and just subcutaneous injections of ixekizumab, at a dilution of one-one hundredth of the systemic dose, could have a therapeutic effect. Evidence from this case suggests that subcutaneous injections near joint lesions may also benefit those with other forms of inflammatory arthritis.

As this was a case study, it is hard for us to compare the efficacy of systematic treatments, but the initial evidence was that local injections in combination with oral tofacitinib had a satisfactory effect in a relatively short period of time. For this patient, who had experienced clear systemic side effects and a limited improvement after several initial treatments, the targeted localized use of therapeutic antibodies was more acceptable and effective. Therefore, this report provides an initial evidence for further randomized controlled trials. However, protocols must intercalate quantitative assessments of chronic inflammation by monitoring changes in the spleen volume^[6]^. The long-term, large-scale studies warrant in the future.

Special thanks to our patient who agreed to participate and for consenting to publish this report. This work was supported by the National Natural Science Foundation of China (Grant No. 82073439).

Yours Sincerely,Ruiyuan Xia^1,△^, Weixin Zhang^2,△^, Jing Hang^2,✉^, Zhiqiang Yin^1,✉^ ^1^Department of Dermatology,the First Affiliated Hospital of Nanjing Medical University,Nanjing, Jiangsu 210029,China;^2^Department of Ultrasound,the First Affiliated Hospital of Nanjing Medical University,Nanjing, Jiangsu 210029,China.^△^These authors contributed equally to this work.^✉^Corresponding authors: Zhiqiang Yin and Jing Hang. E-mails: yinzhiqiang@njmu.edu.cn (Yin) and hangjing@jsph.org.cn (Hang).
